# A secondary EWMA-based dictionary learning algorithm for polynomial phase signal denoising

**DOI:** 10.1038/s41598-022-16644-y

**Published:** 2022-08-20

**Authors:** Guojian Ou, Sai Zou, Song Liu, Jianguo Tang

**Affiliations:** 1 Xichang University, Xichang, 615000 Sichuan China; 2 Guizhou University, Guiyang, 550025 Guizhou China; 3 Chongqing University of Posts and Telecommunications, Chongqing, 400001 China; 4 Chongqing College of Electronic Engineering, Chongqing, 401331 China

**Keywords:** Engineering, Electronics, photonics and device physics, Information theory and computation, Techniques and instrumentation

## Abstract

Under the influence of additive white Gaussian noise, sparse representation cannot effectively remove noise associated with the polynomial phase signal (PPS) via most dictionary learning algorithms whose training data come from the noisy signal, such as K-SVD and RLS-DLA. In this paper, we present a novel dictionary learning algorithm based on secondary exponentially weighted moving average (SEWMA) to denoise PPS. In the proposed algorithm, we first estimate the signal-to-noise (SNR) of the PPS to set the optimal rate of a weighted decline using covariance matrix model. Second we use RLS-DLA to train the dictionary. Thirdly, SEWMA is used to refine atoms in the learned dictionary. In this way, the SNR of the reconstructed signal obtained using the proposed algorithm is clearly higher than that of other algorithms, whereas the mean squared error is lower than that of other algorithms. To obtain the optimal denoising performance, the optimal rate of a weighted decline is set based on the estimated SNR. Simulation results show that the proposed method outperforms the K-SVD, RLS-DLA in mean square error and the SNR.

## Introduction

The polynomial phase signal (PPS) denoising problem is important to improve PPS quality, not only because of the evident applications it serves. In the scientific research and practical applications, it is important to preprocess the signals before the signals are processed, and the most important is to eliminate the noise of the signals. In order to eliminate the noise of the signals, many methods are presented. In these methods, sparse representation is a effective method to denoise the signal. However, the denoising effect is related to the dictionary using sparse representation to denoising the signal. Thus we need to obtain an adaptive dictionary using learning method, and it is described as dictionary learning.

Dictionary learning has broad applications in characteristic extraction, signal reconstruction, signal denoising, compression sensing, and classification^1^, and it provides a novel way to reduce the number of atoms in the over-completed dictionary. This method can cause the learned dictionary to gather information about the dictionary’s content, features, or texture that is more consistent with the signal. In addition, prior information does not need to be applied in dictionary learning, and a better representation of many types of signals can be obtained^2^. These dictionary learning algorithms convey an optimal representation of sparse representation from data, which ensures that atomic scales and characteristics more closely approximate the signal to be represented. Many dictionary learning algorithms have emerged to accomplish these objectives, such as K-SVD^3,4^, RLS-DLA^5^, WTI-kSVD^1^, SBD-STM^6^ and SBL-DF^7^. Among these dictionary learning algorithms, sparse decomposition and dictionary update are two contents of the main research. Although dictionaries obtained using dictionary learning algorithms exhibit a denoising effect via sparse representation, the design of these algorithms to facilitate efficient signal denoising remains a focus of dictionary learning research.

Among dictionary learning algorithms for signal denoising, image denoising algorithms are most commonly studied. Image optimization of image via a learned dictionary is mainly considered using these algorithms, wherein the dictionary learns from external data or a noisy image itself to achieve image denoising^8–12^. Some other denoising algorithms, such as data-driven non-negative dictionary learning, are applied to denoise the seismic data^13,14^. Instead of analytical dictionaries, data-driven dictionary learning methods as a flexible framework for sparse representation, are dedicated to the problem of denoising and interpolation. The atoms in learned dictionaries, however, are not analyzed with these algorithms among these algorithms, nor are they optimized. Although sparse representation exerts a denoising effect on image signals when using a learned dictionary, the effect is not especially noticeable when denoising PPS.

The polynomial phase signal (PPS) has broad applications in radar, sonar, communication, and various nature signal analysis domains^15–18^; therefore, estimating PPS parameters has important theoretical significance and practical value, and several theories and estimation algorithms have been proposed in this vein. However, many denoising methods, such as wavelet denoising method and SVD denoising method, are not suitable for denoising PPS because the frequency of PPS varies according to time. Sparse representation using an over-completed dictionary constructed of time–frequency atoms can denoise PPS, yet the computational complexity is high given the vast number of atoms in the dictionary. Although the intelligent algorithms can reduce this computational complexity^19–22^, they contain a certain degree of randomness that may be infeasible in specific applications. Other researchers have suggested fast sparse decomposition algorithms based on the structural properties of atomic libraries, such as algorithms based on the core atomic libraryand those based on the partitioned concentration^23–25^. Although these fast algorithms contain no randomness, the decomposition rates of these algorithms also need to be further improved.

To effectively remove noise from PPS, a dictionary learning algorithm based on a secondary exponentially weighted moving average (SEWMA) is proposed in this paper. We used the noisy PPS as training data in the proposed algorithm, which we divide into two steps. In the first step, RLS-DLA is used to train the dictionary, whose convergence is better than K-SVD. In the second step, each atom in the dictionary obtained in the first step is optimized via the SEWMA, such that atoms in the dictionary can reduce the noisy component. Accordingly, PPS noise can be effectively removed via sparse representation over the learned dictionary.

The algorithm proposed in this paper is novel in that it optimizes a trained dictionary by using a SEWMA, enabling atoms in the dictionary to be de-noised. In so doing, we can effectively denoise PPS via sparse representation over the learned dictionary. At the same time, the stability of the proposed algorithm is better than the nonlinear least squares (NLLS) method because it has no use for the initial values, yet the NLLS method depends on the initial values. In terms of the computational complexity, the proposed algorithm is less than the NLLS method. In addition, due to use the PPS embedded in noisy that needs to be denoised as training data, we use phase-space reconstruction method to obtain the training data.

The remainder of this paper is organized as follows. First, a brief introduction to relevant dictionary learning algorithms, and phase space reconstruction theory is provided in “Related work” section. Second, our dictionary learning denoising algorithm based on a SEWMA is proposed in “The proposed algorithm” section. Finally, “Experiments” section presents simulations that verify the excellent denoising performance of the method.

## Related work

In this section, we provide an overview of dictionary learning, EWMA, and phase space reconstruction theory.

### Dictionary learning algorithms

Dictionary learning algorithms train a dictionary using a series of training data to minimize the sparse representation error of the training signal based on the learned dictionary, thus rendering the trained dictionary more adaptable to the problems to be solved. Therefore, for a matrix ${\mathbf{Y}} \in {\mathbf{R}}^{N \times L}$ composed of a series of training signals ${\mathbf{y}}_{i} \in {\mathbf{R}}^{N} ,\;i = 1,2, \ldots ,L$, dictionary learning algorithms should solve such problems:

1 $$\begin{aligned} & \mathop {\arg \min }\limits_{D,W} \;f(D,W) = \mathop {\arg \min }\limits_{D,W} \mathop {\left\| {{\mathbf{Y - DW}}} \right\|}\nolimits_{F}^{2} \, \\ \, & \quad s.t. \, \;\;\forall 1 \le i \le L,\;\left\| {w_{i} } \right\|_{0} \le s \\ \end{aligned}$$

where ${\mathbf{W}} \in {\mathbf{R}}^{K \times L}$ is a coefficient matrix, ${\mathbf{D}} \in {\mathbf{R}}^{N \times K}$ denotes a dictionary that requires training, $s$ denotes a sparse constraint and, $f( \cdot )$ denotes a cost function. Researchers have proposed many dictionary learning algorithms to address this problem, which are divided into three main types: probability-based learning algorithms, learning algorithms based on clustering or vector quantization, and structure-based learning algorithms^3^. K-SVD belongs to the second dictionary learning algorithm, which achieves the dictionary learning using SVD method.

To improve the convergence and efficiency of the dictionary learning algorithm compared to K-SVD, k.skretting and k.engan proposed an RLS-DLA dictionary learning algorithm that uses the recursive least squares method to learn the dictionary^5^:

2 $${\mathbf{D}}_{i} = ({\mathbf{Y}}_{i} {\mathbf{W}}_{i}^{T} )({\mathbf{W}}_{i} {\mathbf{W}}_{i}^{T} )^{ - 1} { = }{\mathbf{B}}_{i} {\mathbf{C}}_{i}$$

where ${\mathbf{Y}}_{i} { = }\left[ {y_{1} ,y_{2} , \ldots ,y_{i} } \right] \in {\mathbf{R}}^{N \times i},$ ${\mathbf{W}}_{i} { = }\left[ {w_{1} ,w_{2} , \ldots ,w_{i} } \right] \in {\mathbf{R}}^{K \times i},$ ${\mathbf{B}}_{i} { = }{\mathbf{Y}}_{i} {\mathbf{W}}_{i}^{T} { = }\sum\nolimits_{j = 1}^{i} {y_{j} w_{j}^{T}},$ ${\mathbf{C}}_{i} { = }{\mathbf{W}}_{i} {\mathbf{W}}_{i}^{T} { = }\sum\nolimits_{j = 1}^{i} {w_{j} w_{j}^{T} }.$ According to the inverse lemma and the forgetting factor introduced by the matrix, the formula for the RLS-DLA algorithm is as follows:

3 $$\left\{ {\begin{array}{*{20}l} {C_{i + 1} = (\lambda^{ - 1} C_{i} ) - \alpha uu^{T} } \hfill \\ {D_{i + 1} = D_{i} + \alpha ru^{T} } \hfill \\ \end{array} } \right.$$

where $u = (\lambda^{ - 1} C_{i} )w$, $\alpha = \frac{1}{{1 + w^{T} u}}$, $$\lambda_{i} = \left\{ {\begin{array}{*{20}l} {1 - (1 - \lambda_{0} )(1 - i/a)^{p} } \hfill & {{\text{if}}\;i \le a} \hfill \\ 1 \hfill & {{\text{if}}\;i > a} \hfill \\ \end{array} } \right.$$, Therefore, RLS-DLA completes the dictionary learning iteratively as shown above.

The preceding two dictionary learning algorithms indicate that such algorithms are divided into two stages: a dictionary update stage and a sparse decomposition stage. Thus, these algorithms are improved via these stages, including through multiple dictionary updates and coefficient reuse^26^, the multiple clusters pursuit proposed in^27^, and the deep dictionary learning algorithm presented in^28^.

For the dictionary learning algorithm of signal denoising, trained data can be divided into two types^10^: data without noise, whose dictionary learning algorithm is known as an external prior dictionary learning algorithm; and data from the noisy signal itself, whose corresponding algorithm is labeled an internal prior dictionary learning algorithm. The external prior dictionary learning algorithm may not be adaptive to the signal to be de-noised, and fine-scale image structures may not be recovered well. Although the internal prior dictionary learning algorithm is highly adaptive, it is greatly affected by noise. According to the problems to be solved in (1) and (2), a learned dictionary is related to trained data, and trained data with noise make the trained atom noisy; therefore, using such a learned dictionary for signal denoising must be less effective than the learned dictionary developed based on external data. Thus, reducing the learning dictionary’s atomic noise will inevitably improve the denoising effect of the signal.

### EWMA and its analysis

EWMA is a way to denoise the noisy signal. This method can cause the weighted coefficient of each observation to be decreased exponentially with time. The closer it is to the value of the current moment, the larger the weighted coefficient is. EWMA is adopted because the recent observations of the observation period will greatly affect the predicted value, and it better reflects the trend of recent changes.

EWMA is evolved from weighted moving average (WMA), where the weighted coefficients of each value decrease exponentially over time, the greater the closer to the current moment. Comparing the traditional average method, it does not need to save all the past values, and its computation complexity is significant reduced.

The EWMA formula is expressed as:

4 $$\left\{ {\begin{array}{*{20}l} {y^{\prime}\left( n \right) = \left( {1 - e} \right)y^{\prime}\left( {n - 1} \right) + ey\left( n \right)} \hfill \\ {y^{\prime}\left( 0 \right) = y\left( 0 \right)} \hfill \\ \end{array} } \right.\quad n \in [1,N]$$

where $y^{\prime}\left( n \right)$ denotes the predicted value at the current moment $n$, $y^{\prime}\left( {n - 1} \right)$ denotes the predicted value at last moment $n - 1$, $e$ is the rate of a weighted decline, which is a constant that the smaller the value, the faster the weight drops, $y^{\prime}\left( 0 \right)$ denotes the initial value $y\left( 0 \right)$.

The EWMA can be expressed in another way as

5 $$\left\{ {\begin{array}{*{20}l} {y^{\prime}\left( 0 \right) = y\left( 0 \right)} \hfill \\ {y^{\prime}\left( 1 \right) = \left( {1 - e} \right)y\left( 0 \right) + ey\left( 1 \right)} \hfill \\ {y^{\prime}\left( 2 \right) = \left( {1 - e} \right)^{2} y\left( 0 \right) + e\left( {1 - e} \right)y\left( 1 \right) + ey\left( 2 \right)} \hfill \\ \cdots \hfill \\ {y^{\prime}\left( n \right) = \left( {1 - e} \right)^{n} y\left( 0 \right) + \sum\limits_{i = 1}^{n} {\left[ {e\left( {1 - e} \right)^{n - i} y\left( i \right)} \right]} } \hfill \\ \end{array} } \right.$$

**Figure 1:**
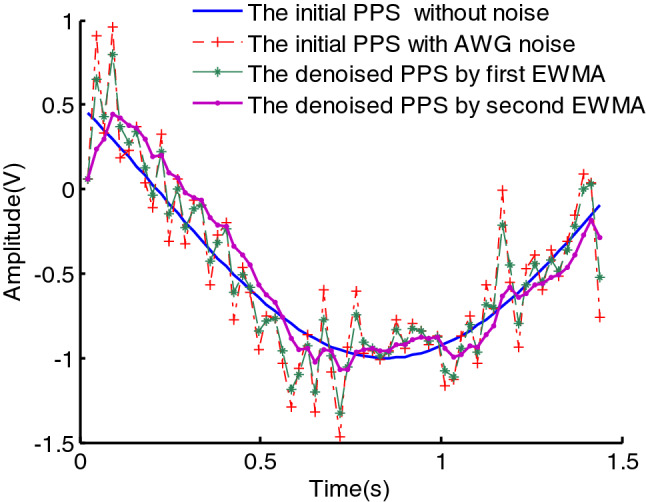
The denoising effect using EWMA.

It can be seen from the above formula, $y^{\prime}\left( n \right)$ sums the data from the past period with different weights. Due to $e < 1$, the closer to the current moment, the greater the weight of $y\left( i \right)$. However, because $y\left( n \right)$ is a noisy data, the denoising effect achieved by the first EWMA is poor and the smoothing characteristic is not good, as can be seen in Fig. 1. In this figure,$y\left( n \right)$ is set as

6 $$y(n) = \cos \left( {\sum\limits_{m = 0}^{3} {a_{m} n^{m} } } \right) + \upsilon (n),\quad n \in [0,N]$$

where $a = \left( {a_{3} \;\;a_{2} \;\;a_{1} \;\;a_{0} } \right) = \left( {2.75e - 6 \, 1.25e - 3\pi {/}8\pi {/}3} \right)$, $\upsilon (n)$ is white Gaussian noise of power, $\sigma^{2}$,the SNR is set as $12\;{\text{dB}}$, the number of observation $N{ = }64$, the rate of a weighted decline $e = 0.7$.It can be seen in Fig. 1 that the denoising effect achieved using the first EWMA is poor, but the effect and the smoothing characteristic are better using the second EWMA.

**Figure 2:**
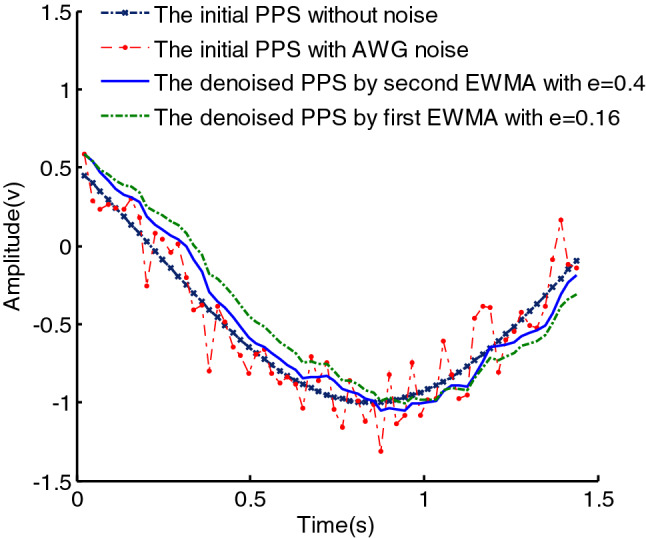
The denoising effects using the first EWMA and the second EWMA.

We can judge that the second EWMA will improve the denoising effect, which is also seen from formula (7). Since the second EWMA is used, the rate of a weighted decline has been changed from $e$ to $e^{2}$. Owing to $e < 1$, the second EWMA makes the rate of a weighted decline smaller, and the smoothing characteristic of the signal is better. For example, we set the rate of a weighted decline as 0.4 in the first EWMA, thus the rate of a weighted decline in the second EWMA is changed as $0.4^{2}$. But the denoising effect over $e = 0.16$ in the first EWMA and $e = 0.4$ in the second EWMA is different, which can be seen as Fig. 2. We can see that the smoothing characteristic of the signal using the second EWMA is basically the same as the first EWMA, but the reconstructed PPS using the second EWMA is closer to the noise-free PPS than the first EWMA.

7 $$\left\{ {\begin{array}{*{20}l} {y^{\prime\prime}\left( 0 \right) = y^{\prime}\left( 0 \right)} \hfill \\ { = y\left( 0 \right)} \hfill \\ {y^{\prime\prime}\left( 1 \right) = \left( {1 - e} \right)y^{\prime\prime}\left( 0 \right) + ey^{\prime}\left( 1 \right)} \hfill \\ { = \left( {1 - e} \right)\left( {1 + e} \right)y\left( 0 \right) + e^{2} y\left( 1 \right)} \hfill \\ {y^{\prime\prime}\left( 2 \right) = \left( {1 - e} \right)y^{\prime\prime}\left( 1 \right) + ey^{\prime}\left( 2 \right)} \hfill \\ { = \left( {1 - e} \right)^{2} \left( {1 + 2e} \right)y\left( 0 \right) + 2\left( {1 - e} \right)e^{2} y\left( 1 \right) + e^{2} y\left( 2 \right)} \hfill \\ \cdots \hfill \\ {y^{\prime\prime}\left( n \right) = \left( {1 - e} \right)y^{\prime\prime}\left( {n - 1} \right) + ey^{\prime}\left( n \right)} \hfill \\ { = \left( {1 - e} \right)^{n} \left( {1 + ne} \right)y\left( 0 \right) + \sum\limits_{i = 1}^{n} {\left[ {\left( {n - i + 1} \right)e^{2} \left( {1 - e} \right)^{n - i} y\left( i \right)} \right]} } \hfill \\ \end{array} } \right.\quad n \in [1,N]$$

**Figure 3:**
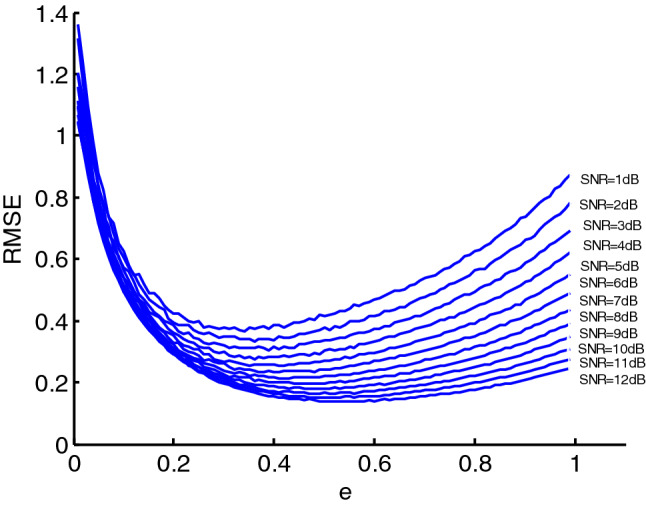
The RMSE of PPS over different e.

**Figure 4:**
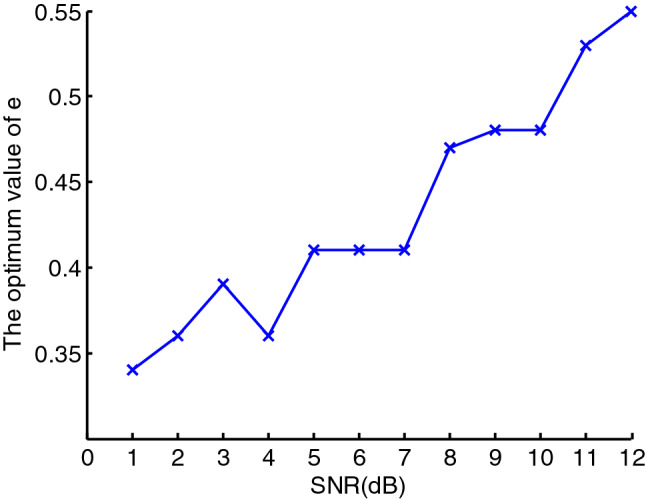
The optimum value of e over the SNR from 1 to 12 dB.

As can be seen from the formula (4), the denoising effect using EWMA is closely related to the rate of a weighted decline $e$. The smaller $e$ is, the smoother the reconstructed signal, however, the smaller $e$ cannot guarantees that the denoising effect is better. Therefore setting the value of $e$ is of great significance for the denoising effect, and the optimal value of $e$ is closely related to the SNR, which can be seen as Figs. 3 and 4. In the two Figures, the SNR of PPS is set from $1$ to $12\;{\text{dB}}$, and the figure shows that with the increase of the SNR, the optimal value of $e$ corresponding to the minimum value of the root mean square error (RMSE) also increase. Therefore, to achieve the optimal denoising effect, the optimal value of $e$ should be taken according to the SNR.

### Blind SNR estimation of PPS

According to the above analysis, we need to estimate the SNR of the PPS to obtain the optimal value of $e$. Actually, we need to do blind SNR estimation over the PPS. Blind SNR estimation technology is an important technology in signal processing. In many cases, SNR is given as prior information and directly affects the selection and analysis results of signal processing methods.

The main SNR estimation algorithms include the maximum-likelihood estimation method, spectral analysis-based estimation method and statistics-based estimation method^29^, which can also be divided into data-assisted estimation, judgment guidance estimation and non-data-assisted estimation^30^. But many of these algorithms are not suitable for the SNR estimation of PPS, so it is necessary to design a SNR method conducive to the SNR estimation of PPS.

Here, according to the characteristics of PPS, we employ the SVD of the covariance matrix to estimate the SNR of PPS, which has better performance than using the autocorrelation matrix, as follow:

Set PPS as

8 $$y(n) = b\cos \left( {j\sum\limits_{m = 0}^{P} {a_{m} } n^{m} } \right) + \upsilon (n)\quad n \in \left[ {1,N} \right]$$

where $b$ and $a_{m}$ are the coefficients of the PPS, $\upsilon (n)$ is white Gaussian noise of power, $\sigma^{2}$,thus the covariance matrix can be expressed as

9 $$r\left( {l,k} \right) = \frac{1}{N - Q}\sum\limits_{i = 1}^{N - Q} {y\left( {i + l} \right)} y^{*} \left( {i + k} \right)\quad l,k = 0,1,2, \ldots Q$$

So It is expressed in another way as

10 $${\mathbf{R}} = \left[ {\begin{array}{*{20}c} {r\left( {0,0} \right)} & {r\left( {0,1} \right)} & {r\left( {0,Q} \right)} \\ {r\left( {1,0} \right)} & {r\left( {1,1} \right)} & {r\left( {1,Q} \right)} \\ \vdots & \vdots & \vdots \\ {r\left( {Q,0} \right)} & {r\left( {Q,1} \right)} & {r\left( {Q,Q} \right)} \\ \end{array} } \right]$$

Implement the SVD for ${\mathbf{R}}$, so

11 $${\mathbf{R}} = {\mathbf{U\Sigma V}}^{{\text{T}}}$$

where ${{\varvec{\Sigma}}} = diag\left( {\sigma_{1}^{2} + \sigma^{2} ,\sigma_{2}^{2} + \sigma^{2} , \ldots ,\sigma_{r}^{2} + \sigma^{2} ,\sigma^{2} , \ldots ,\sigma^{2} } \right)$, $\sigma_{1}^{2} \ge \sigma_{2}^{2} \ge \cdots \sigma_{r}^{2}$. According to the analysis over SVD, we can see that the variety of the tangent between $\sigma_{r}^{2} + \sigma^{2}$ and $\sigma^{2}$ is maximal, thus the number $N_{s}$ from $\sigma_{1}^{2} + \sigma^{2}$ to $\sigma_{r}^{2} + \sigma^{2}$ can be determined, and the number $Q - N_{s}$ of $\sigma^{2}$ item can be also determined. So the SNR of the PPS can be expressed as

12 $$SNR = \frac{{N_{s} \left( {\sigma_{r}^{2} + \sigma^{2} } \right) - \left( {Q - N_{s} } \right)\sigma^{2} }}{{\left( {Q - N_{s} } \right)\sigma^{2} }}$$

### Phase space reconstruction theory

The phase space is generally considered multidimensional, and its goal is to describe all possible states of certain dynamic systems from a mathematical and physical point of view^31^. The core objective of phase space reconstruction is to map a low-dimensional time series to a high-dimensional phase space. After this transformation, the dynamic properties of the time series remain unchanged.

According to phase space reconstruction theory, the evolution of any system component is determined by the other components that interact with it. Therefore, all system information is implied in the evolution of each component. When reconstructing a state space, only one component is considered; this component is treated as a new dimension at some fixed time delay point. In addition, the noisy signal $y\left( n \right)$,$n = 1,2, \ldots ,N$ measured from a system can be embedded into the attractor orbit matrix ${\mathbf{D}}_{n}$ with the number of the dimensions $L$ based on phase space reconstruction theory. ${\mathbf{D}}_{n}$ is expressed as follows:

13 $${\mathbf{D}}_{n} = \left[ {\begin{array}{*{20}c} {y_{1} } & {y_{2} } & \cdots & {y_{N - L + 1} } \\ {y_{2} } & {y_{3} } & \cdots & {y_{N - L + 2} } \\ \vdots & \vdots & \ddots & \vdots \\ {y_{L} } & {y_{L + 1} } & \cdots & {y_{N} } \\ \end{array} } \right]$$

From (13) ${\mathbf{D}}_{n}$ may be written as ${\mathbf{D}}_{n} = {\mathbf{D + \upsilon }}$, where ${\mathbf{D}}$ is a $m \times n$ dimension matrix composed of signals without noise, and ${{\varvec{\upupsilon}}}$ is a $m \times n$ dimension matrix consisting of noise.

## The proposed algorithm

In this section, we detail our dictionary learning algorithm for PPS denoising based on a second EWMA, which is referred to as SEWMA-DLA. In this algorithm, we first estimate the SNR of the PPS to achieve the optimal denoising effect, and the optimal $e$ can be obtained based on the estimated SNR. By optimizing atoms in the learned dictionary and sparse representation, PPS is effectively de-noised.

### Atoms in learned dictionary

From the above analysis, it is clear that the training data of dictionary learning can be divided into two categories: the data without noise and the object signal with noise. The former is not adaptive, and the latter is adaptive, but it is greatly affected by noise. For the latter, this main reason is that the learned dictionary necessarily contains the noise component when the dictionary is trained using noise signal as training data. Therefore, atoms in the dictionary obtained by RLS-DLA contain the same noise. The issue of interest in this dictionary learning algorithm is $\min \left\| {{\mathbf{Y - DW}}} \right\|_{F}^{2}$, and the learned dictionary ${\mathbf{D}} = ({\mathbf{YW}}^{T} )({\mathbf{WW}}^{T} )^{ - 1}$; thus, the atoms of ${\mathbf{D}}$ contain the same noise as in the signal matrix ${\mathbf{Y}}$,and the atom is a combination of a range of training data, whose detailed derivation is given in the Appendix.

**Figure 5:**
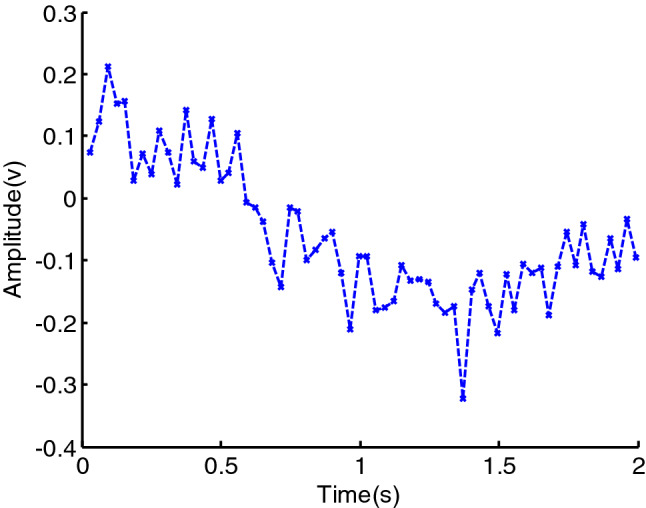
Curve of one atom in the dictionary learned by RLS-DLA.

In fact, whether using the RLS-DLA dictionary learning algorithm, K-SVD algorithm, or another dictionary learning algorithm, as long as the training data contain noise, the atoms in the learned dictionary must also contain noise, which can be verified by an experiment. We set the real third-order PPS as follows^32^:

14 $$y(n) = b\cos \left( {\sum\limits_{i = 0}^{3} {a_{i} (n)^{i} } } \right) + \upsilon (n),\quad n \in [0,N]$$

where $a = \left( {a_{3} \;\;a_{2} \;\;a_{1} \;\;a_{0} } \right) = \left( {1.75e^{ - 6} \, 1.05e^{ - 3} \, \frac{\pi }{10} \, \frac{\pi }{3}} \right)$, $b{ = }1$; $\upsilon (n)$ is white Gaussian noise of power, $\sigma^{2}$, and the SNR is set to $10\;{\text{dB}}$. RLS-DLA is used to train the dictionary. During training, the third-order PPS with noise constitutes training data. One atom in the learned dictionary is displayed in Fig. 5. Atoms in the learned dictionary represent a function with additive white Gaussian noise, and the denoising effect is not especially obvious when using these atoms.

**Figure 6:**
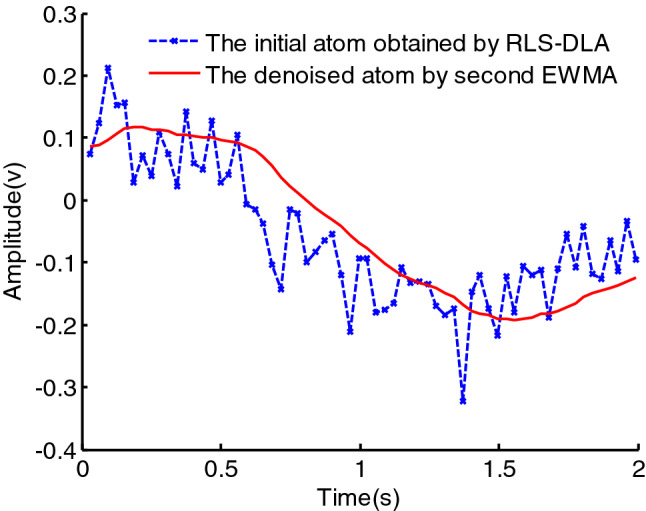
Atom in learned dictionary.

If the problem of atomic noise can be solved, then signal denoising will become much more efficient. Therefore, we use a second EWMA to denoise atoms with noise, after which signal denoising can be completed via sparse representation. Figure 6 displays the process of denoising one atom using a second EWMA. There is additive white Gaussian noise in the atom in the dictionary learned via RLS-DLA, whereas the atom processed by the second EWMA is a smooth curve. Clearly, the second EWMA effectively removes atomic noise. The sparse representation of the signal can thus greatly improve the denoising efficiency of the signal by using the learned dictionary processed via the second EWMA.

It should be noted here that the signal denoising effect is related to $e$, and setting the value of $e$ needs to obtain the SNR of the signal, thus we need to obtain the SNR via relevant algorithms.

### SEWMA-DLA algorithm

According to the above analysis, we propose a dictionary learning algorithm based on direct weight determination of a neural network for PPS denoising, called SEWMA_DLA. In this algorithm, we first need to reconstruct covariance matrix, then implement SVD to estimate the SNR of PPS, thus the optimal $e$ can be set by the estimated SNR. For the initial dictionary, it is expressed as ${\mathbf{D}}_{0} \in {\text{R}}^{N \times K}$, each column of the dictionary denotes an atom, and $K$ is the number of atoms.

As a redundant dictionary is being adopted, $K > > N$,${\mathbf{Y}} \in {\mathbf{R}}^{N \times L}$ denotes the set of trained signals, where $L < < K$; thus, the coefficient matrix is ${\mathbf{W}} \in {\mathbf{R}}^{K \times L}$. Because the signal to be denoised serves as training data, the length of the signal must be considered. During PPS processing, the signal length is not particularly long. However, the number $L$ of the trained signal is much larger than the number of atoms in the redundant dictionary. If the object signal $y\left( i \right)\left( {i = 1,2, \ldots ,M} \right)$ is not long enough, then based on general segmentation conditions, the signal $y\left( i \right)$ whose length is $M$ is divided into $L$ segments using the discrete time order. The set of trained signals ${\mathbf{Y}}$ can then be obtained. The number $L$ of trained signals cannot be satisfied with $L > > K$, and $L < K$ is possible. To satisfy this condition, we need to reconstruct the training signal set ${\mathbf{Y}}$ based on phase space reconstruction theory^33^:

15 $${\mathbf{Y}}{ = }\left[ {\begin{array}{*{20}c} {y_{1} } & {y_{r + 1} } & \cdots & {y_{{r*\left( {L - 1} \right) + 1}} } \\ {y_{2} } & {y_{r + 2} } & \cdots & {y_{{r*\left( {L - 1} \right) + 2}} } \\ \vdots & \vdots & \ddots & \vdots \\ {y_{N} } & {y_{N + r} } & \cdots & {y_{{r*\left( {L - 1} \right) + N}} } \\ \end{array} } \right]$$

where $N$ is the length of each trained signal, $r < < N$,$r$,$M$ and $L$ are satisfied with $r*\left( {L - 1} \right) + N = M$. As such, $L$ is satisfied with $L > > K$ through the above processing method, and dictionary learning can be achieved using the proposed algorithm. Next, we can use the second EWMA to denoise the atoms in the learned dictionary, and PPS can be de-noised via sparse representation over the learned dictionary whose atoms are de-noised. The specific algorithm is described as follows:


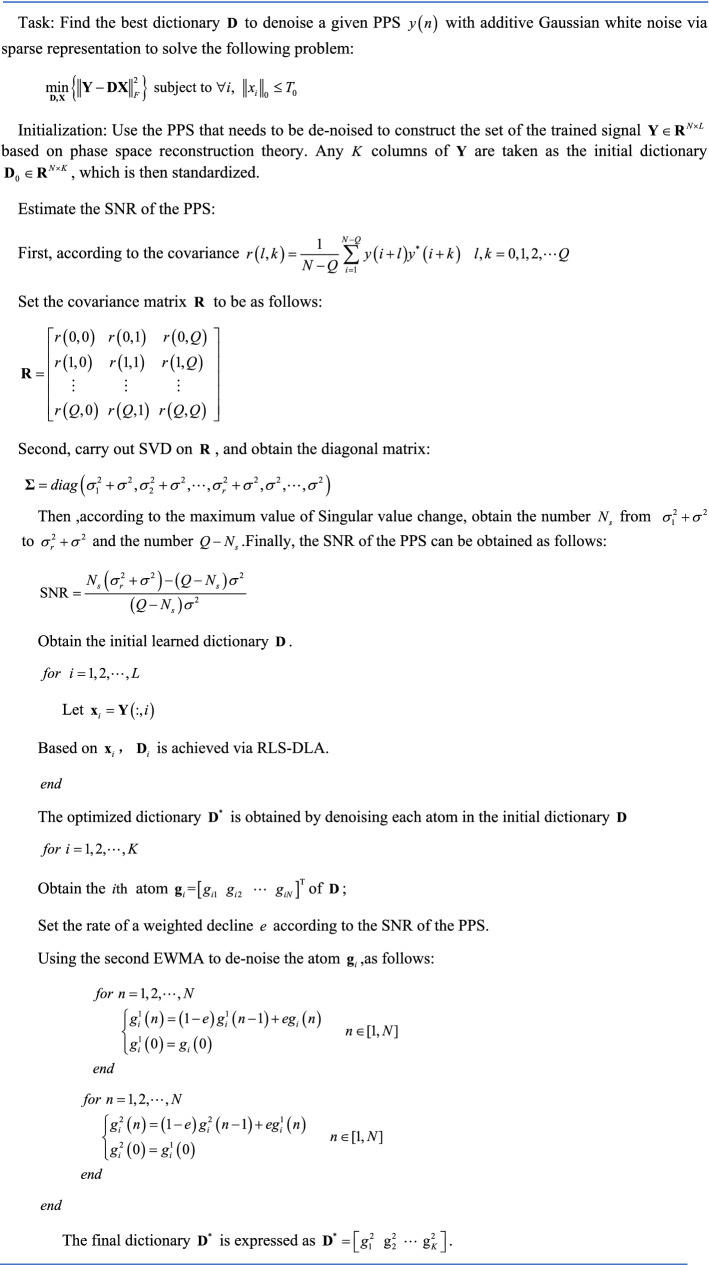


### Analysis of algorithm

In the proposed SEWMA_DLA algorithm, RLS-DLA is used to obtain the initial learned dictionary in the first step, after which we use the second EWMA to improve atoms in the initial learned dictionary. In the second step, we estimate the SNR of the PPS to set the optimal $e$. In the first step the main reasons for using the RLS-DLA is that it can achieve global convergence in the training dictionary, and it demonstrates good convergence properties and can avoid local convergence of K-SVD and other algorithms. In addition, due to introducing the forgetting factor $\lambda$, RLS-DLA relies less on the initial dictionary and can improve the algorithm’s convergence. The computational complexity of RLS-DLA is $O\left( {\left( {s^{2} + N + K} \right)KL} \right)$^5^, where $s$ is the sparseness, $N$ is the length of atoms or the length of training signals, $K$ is the number of atoms in the learned dictionary, and $L$ is the number of training signals.

We use the second EWMA to denoise the atoms in the SEWMA_DLA algorithm, and the computational complexity of the EWMA is $O\left( N \right)$. Since there are $K$ atoms in the learned dictionary, the computational complexity is $O\left( {KN} \right)$. In addition, since we use SVD to estimate the SNR, the computational complexity of SEWMA_DLA algorithm is related to the SVD. The computational complexity of the SVD is related to the dimension $m$ of the matrix, which is expressed as $O\left( {m^{3} } \right)$^34^. $m$ is generally set to 50–100. If the selection of $m$ is too small, matrix distortion is large, which will affect the estimation of the SNR. Conversely, if the selection of $m$ is too large, unnecessary calculations will be increased.

The preceding analysis indicates that the computational complexity of the proposed algorithm is $O\left( {\left( {s^{2} + N + K} \right)KL + KN + m^{3} } \right)$, $s < N < K \ll L$, where $N$ is the atom length or the training signal length, which is a small number, it can be set to between dozens and 200, such as $N = 64$.

Because $n$ denotes the order of the power function or the order of PPS, it is a relatively small number. In addition, $s < N < K \ll L$; therefore, the complexity of the proposed algorithm is mainly determined by the first step. The computing time in the second step accounts for a small proportion of the proposed algorithm’s entire computing time. From the formula for the computational complexity of the entire algorithm, since $\left( {s^{2} + N + K} \right)KL + KN > > m^{3}$, the computational complexity of the entire algorithm is mainly determined by the second step and the third step, and the computing time in the first step accounts for a small proportion of the proposed algorithm’s entire computing time.

## Experiments

In this section, we demonstrate the denoising effect of the proposed algorithm on PPS. In addition, we compare the computational complexity and convergence with that of the fixed dictionary. The signal denoising effect is generally measured based on the mean squared error (MSE) and SNR, each defined as follows:

16 $$\left\{ {\begin{array}{*{20}l} {{\text{MSE}} = \frac{1}{N}\sum\limits_{n = 1}^{N} {(x(n) - \widehat{x}(n))^{2} } } \hfill \\ {{\text{SNR}} = 10\log \left[ {\frac{{\sum\limits_{n = 1}^{N} {x^{2} (n)} }}{{\sum\limits_{n = 1}^{N} {(x(n) - \widehat{x}(n))^{2} } }}} \right]} \hfill \\ \end{array} } \right.$$

where $N$ is the signal length, $x(k)$ is the $k{\text{th}}$ data point of the signal without noise, and $\widehat{x}(k)$ is the $k{\text{th}}$ data point of the signal with noise. Formula (16) shows that the smaller the MSE, the greater the SNR, and the better the denoising effect. The dictionary learning algorithm proposed in this paper is compared with K-SVD, RLS-DLA, and the denoising K-SVD in^35^ (KSVD_denoising).

Without loss of generality, a real-valued third-order PPS can be expressed as^32^

17 $$y(n) = \cos \left( {\sum\limits_{m = 0}^{3} {a_{m} (n\vartriangle )^{m} } } \right) + \upsilon (n),\quad n \in [0,N]$$

To avoid ambiguity about $a_{m}$ in experiments, we need to limit $a_{m}$ as follows^36^:

18 $$\left| {a_{m} } \right| \le \frac{\pi }{{m!n^{m - 1} \Delta^{m} }}$$

**Figure 7:**
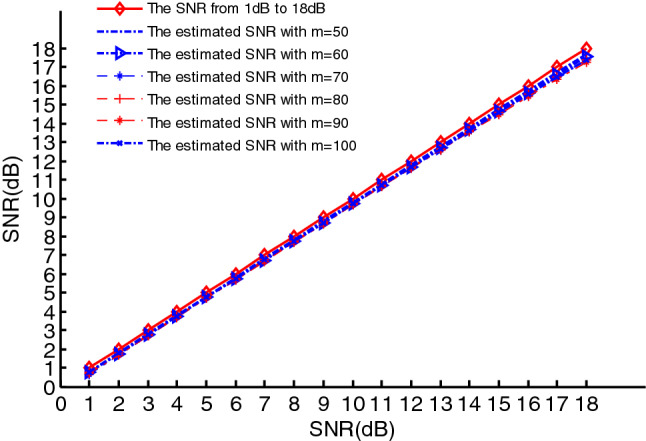
The estimated SNR using the different covariance matrix with different dimensions.

**Figure 8:**
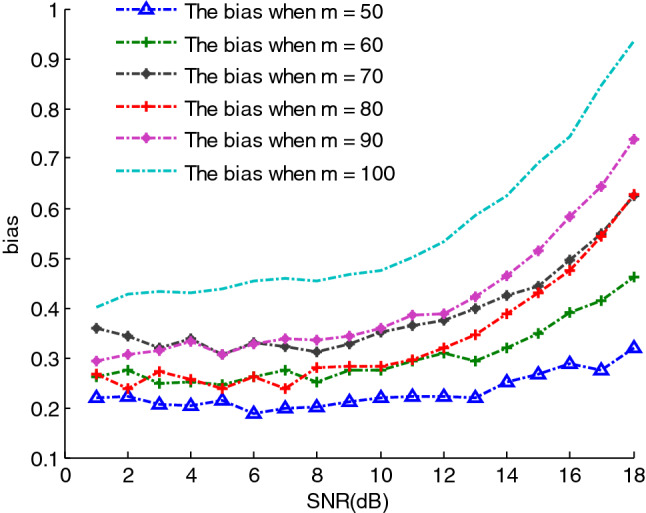
The $bias$ of the estimated SNR using the different covariance matrix with different dimensions.

Therefore, the parameter vector $a$ can be set to $a = \left( {a_{3} \;\;a_{2} \;\;a_{1} \;\;a_{0} } \right) = \left( {2.75e - 6 \, 1.25e - 3 \, \frac{\pi }{8} \, \frac{\pi }{3}} \right)$, $\upsilon (n)$ is additive white Gaussian noise, and the signal observation length $N{ = }1024$. According to SEWMA_DLA algorithm, we first estimate the SNR of the PPS. In order to test the performance of the SNR estimation, the range of the SNR is set from $1$ to $18\;{\text{dB}}$. Meanwhile, to test the effect of covariance dimensions on the estimation performance of the SNR, the range of the dimension $m$ of the covariance matrix is set to $m = 50,60,70,80,90,100$.For each $m$,200 simulations are performed, and the estimated SNRs over different $m$ are shown as Fig. 7. It can be seen that the estimated SNR using the covariance matrix constructed in these six dimensions is relatively close to the actual SNR when $m \in \left[ {50,100} \right]$.For the performance of the SNR estimation, it can be tested using estimated deviation $bias$ and MSE, which are defined as follows:

19 $$\left\{ {\begin{array}{*{20}l} {bias = \left| {\frac{1}{{N_{mc} }}\sum\limits_{i = 1}^{{N_{mc} }} {\rho_{i} - \rho } } \right|} \hfill \\ {{\text{MSE}} = \left( {\frac{1}{{N_{mc} }}\sum\limits_{i = 1}^{{N_{mc} }} {\left( {\rho_{i} - \rho } \right)}^{2} } \right)^{1/2} } \hfill \\ \end{array} } \right.$$

where $N_{mc}$ denotes the number of Monte Carlo experiments, $\rho_{i}$ is the estimated SNR, $\rho$ is the actual SNR. Here, we use $bias$ to test the performance of the SNR estimation, which can be seen as Fig. 8. We can see in the figure that the $bias$ is minimal when $m = 50$, and it is maximal when $m = 100$. Therefore, for a real-valued third-order PPS, the performance of the SNR estimation is best when the dimension of the covariance matrix is 50, which makes the computational complexity minimal.

After estimating the SNR of the PPS, we used RLS-DLA to obtain the initial dictionary. In the step, we used the real-valued third-order PPS with noise as the training signal. The atomic length $M$ is set to 64, the number of atoms $K$ is set to 128, and the sparseness $S$ is set to 16. Considering the signal observation length, the training signal set ${\mathbf{Y}} \in {\mathbf{R}}^{M \times L}$ can be constructed using phase space reconstruction theory:

20 $${\mathbf{Y}} = \left[ {\begin{array}{*{20}c} {y_{1} } & {y_{3} } & \cdots & {y_{{2 \times \left( {r - 1} \right) + 1}} } & \cdots & {y_{{2 \times \left( {L - 1} \right) + 1}} } \\ {y_{2} } & {y_{4} } & \cdots & {y_{{2 \times \left( {r - 1} \right) + 2}} } & \cdots & {y_{{2 \times \left( {L - 1} \right) + 2}} } \\ \vdots & \vdots & {} & \vdots & {} & \vdots \\ {y_{M} } & {y_{2 + M} } & \cdots & {y_{{2 \times \left( {r - 1} \right) + M}} } & \cdots & {y_{{2 \times \left( {L - 1} \right) + M}} } \\ \end{array} } \right]$$

where $r = 1,2, \ldots ,L$, $L = \left( {N - M} \right){/}2 + 1$, $L$ is the number of training signals, set to $L = 960$ based on the above data. Accordingly, $s < M < K < < L$.

**Figure 9:**
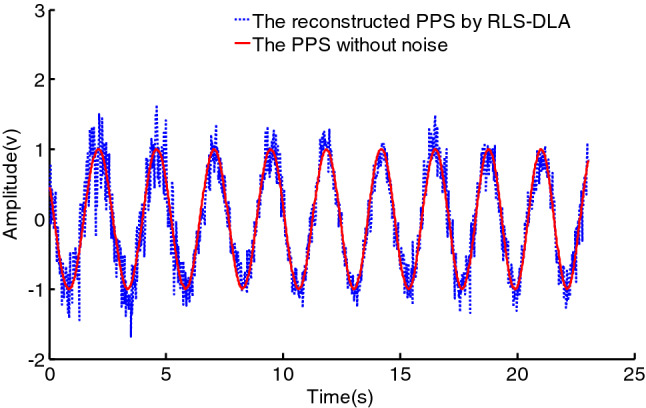
Reconstructed signal by RLS-DLA.

**Figure 10:**
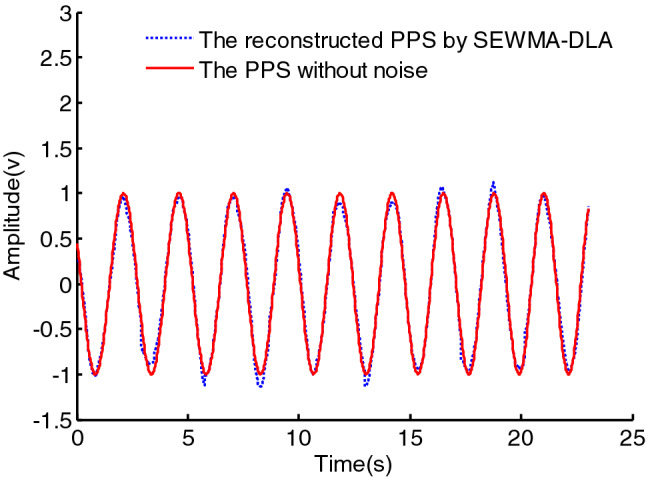
Reconstructed signal by SEWMA-DLA.

In the experiment, the reconstructed signal obtained by sparse representation over the initial dictionary obtaining by the RLS-DLA algorithm is shown in Fig. 9. It can be seen that the reconstructed signal has an obvious noise, and it is obvious that the denoising effect is poor using the RLS-DLA algorithm. This main reason is that the training signal with noise make the learned dictionary contain the same noise as the training signal, which can be seen as Fig. 5. It is obvious that the learned dictionary with noise cannot effectively remove the noise from the signal. The learned dictionary, however, comprises of the atoms de-noised by the second EWMA. The denoised atom is a more smooth curve, which can be seen in Fig. 6. The reconstructed signal is more similar to the PPS without noise via sparse representation over the dictionary trained with the SEWMA_DLA, which can be seen in Fig. 10. We can see that the SEWMA_DLA demonstrates a better denoising effect than the RLS-DLA algorithms.

**Figure 11:**
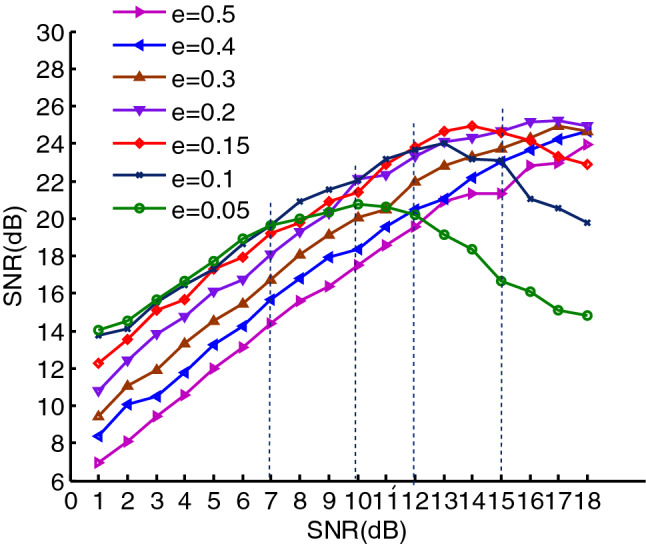
The denoising effects of PPS using different $e$.

**Figure 12:**
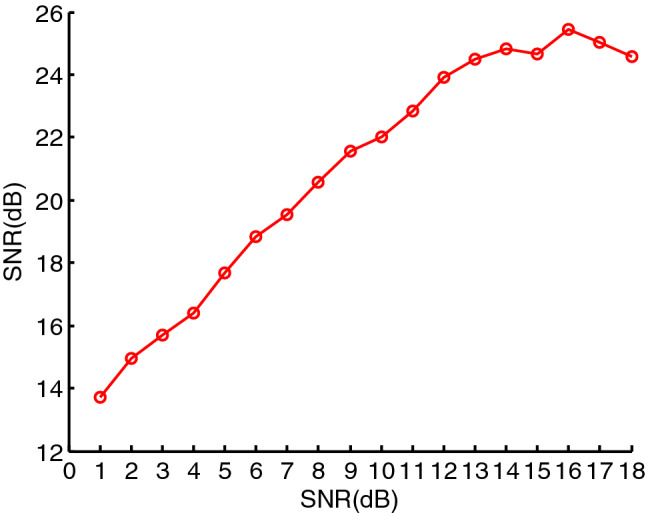
The optimal denoising effect using different $e$.

From the above analysis, the optimal effect of the signal denoising is related to $e$ when EWMA is used to denoise the signal, and $e$ shall be taken based on the SNR, which can be seen in Fig. 11. In the figure, the horizontal axis represents the SNRs before the signal is denoised, whose range is from $1$ to $18\;{\text{dB}}$, and the curves represent the SNRs after the signal is denoised. It is obvious that the smaller the $e$, the better the denoising effect between $1$ and $7\;{\text{dB}}$,thus the denoising effect is best when $e = 0.05$. The denoising effect using the $e$,however, gradually get worse than the other $e$ after the SNR is greater than $7\;{\text{dB}}$, and it is worst when the SNR is greater than $12\;{\text{dB}}$. The denoising effect is best between $7$ and $12\;{\text{dB}}$ when $e = 0.1$, and it best between $12$ and $15\;{\text{dB}}$ when $e = 0.15$. Similarly, it best between $15{\text{dB}}$ to $18{\text{dB}}$ when $e = 0.2$. Therefore, the best denoising effect can be obtain based on $e$, which can be show in Fig. 12. In the figure, since we used the different $e$ at the different SNR stages, the denoising effect of PPS is best.

**Figure 13:**
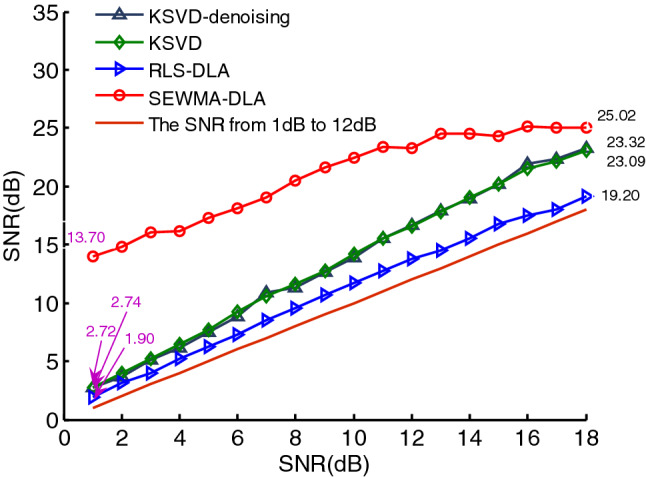
The denoising effect using the four algorithms.

To highlight the denoising effect of the SEWMA-DLA algorithm, here, we compared the denoising effect of the PPS using SEWMA-DLA ,RLS-DLA,K-SVD and KSVD_denoising. The SNRs of the reconstructed PPS using the four algorithms are shown in Fig. 13. We can see that the SNR increases by 0.90–1.74 dB with RLS-DLA, K-SVD, and KSVD_denoising and increase by $12.70\;{\text{dB}}$ using SEWMA-DLA. As the SNR increases to $18\;{\text{dB}}$, the SNR increases by 1.20–5.32 dB under the first three algorithms but increases by ${7}{\text{.02}}\;{\text{dB}}$ under SEWMA-DLA. Evidently, the denoising effect of SEWMA-DLA is more significant compared with the other three algorithms.

**Figure 14:**
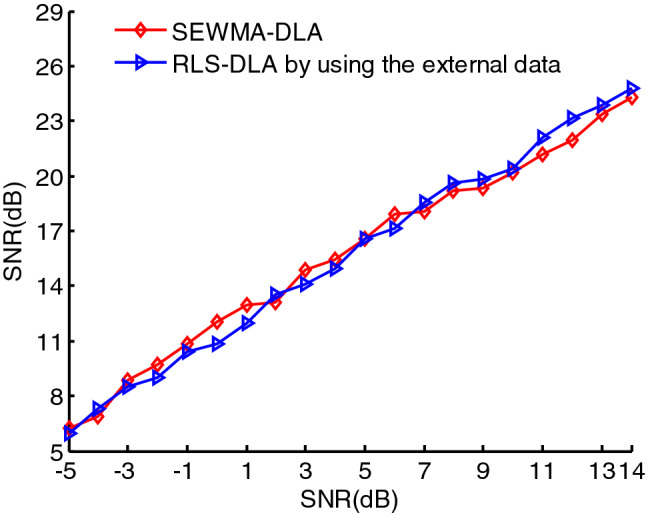
Denoising effects of RLS-DLA using external data and WDNN-DLA using internal data.

To compare the denoising effect based on noise-free external data and internal data with noise, we consider 1000 noise-free PPSs randomly selected from 40,000 first- to fourth-order noise-free PPSs. Then the dictionary can be obtained using RLS-DLA. Here, taking the real-valued third-order PPS mentioned above as an example, the denoising effects based on RLS-DLA using external data and SEWMA-DLA using internal data appear in Fig. 14. The denoising effects are similar when we use two different dictionaries: one obtained via RLS-DLA and external data, and another obtained via SEWMA-DLA and internal data. However, the noisy real-valued third-order PPS itself is used as the training data for SEWMA-DLA, thus, SEWMA-DLA is obviously adaptive.

## Conclusion

In this paper, we proposed a dictionary learning algorithm to denoise PPS, which we named SEWMA-DLA. To denoise PPS, we first took it as training data itself in SEWMA-DLA. Our algorithm was divided into three steps. In the first step, we need to estimate the SNR of the PPS to set the optimal rate of a weighted decline $e$. Then, in the second step, the initial learned dictionary was obtained via RLS-DLA. Finally, the second EWMA was used to refine atoms in the initial learned dictionary. Thus, PPS denoising via sparse representation over the learned dictionary was effectively realized.

From the effect of the experiment, different $e$ leads to different denoising effect of the PPS over the same SNR. Thus we first estimate the SNR of the PPS to realize the best denoising effect in SEWMA-DLA. In addition, we believe that SEWMA-DLA can be used not only for the denoising of the PPS, but also for other signals such as image signals.

## Supplementary Information

Supplementary Information.

## Data Availability

The authors confirm that the data supporting the findings of this study are available within the article or its supplementary materials.
