# Supplementary material for: A secondary EWMA-based dictionary learning algorithm for polynomial phase signal denoising
Source: Sci Rep. 2022 Aug 20;12:14193. doi: 10.1038/s41598-022-16644-y (PMC9392793; doi:10.1038/s41598-022-16644-y)
Supplement: Supplementary file 1 — Supplementary Information. [file 41598_2022_16644_MOESM1_ESM.docx]

**Appendix**

Set the noisy signal itself as the training data, and its training set is defined as

$\mathbf{Y}=\left[\mathbf{y}_{\mathbf{1}}\mathbf{ y}_{\mathbf{2}}\mathbf{ }\cdots\mathbf{ y}_{\mathbf{M}}\right]=\left[\begin{matrix} y_{11}\text{ }y_{21}\text{ }\cdots\text{ }y_{M1} \\ y_{12}\text{ }y_{22}\text{ }\cdots\text{ }y_{M2} \\ \text{ }\vdots\text{ }\ddots\text{ }\vdots\\ y_{1N}\text{ }y_{2N}\text{ }\cdots y_{MN} \end{matrix}\right]$ (1)

According to the training set, the problems to be solved in dictionary learning are

$\arg\min_{\mathbf{D,W}} f\left(\mathbf{D,W}\right)=\arg\min_{\mathbf{D,W}} \left\|\mathbf{Y}-\mathbf{DW}\right\|_{F}^{2}$ (2)

When $\mathbf{W}$ is fixed, $\mathbf{D}$ can be obtained as follows:

$\mathbf{D}=(\mathbf{YW})(\mathbf{WW})^{-1}$ (3)

Using the recursive least squares method, set $\mathbf{Y}_{i}=\left[\mathbf{y}_{\mathbf{1}}\mathbf{ y}_{\mathbf{2}}\mathbf{ }\cdots\mathbf{ y}_{i}\right]\in\mathbf{R}^{N\times i}$, $\mathbf{W}_{i}=\left[\mathbf{w}_{\mathbf{1}}\mathbf{ w}_{\mathbf{2}}\mathbf{ }\cdots\mathbf{ w}_{i}\right]\in\mathbf{R}^{K\times i}$. After the $i\text{th}$training is completed, the dictionary .. is expressed as follows:

$\mathbf{D}_{i}=(\mathbf{Y}_{i}\mathbf{W}_{i}^{\text{T}})(\mathbf{W}_{i}\mathbf{W}_{i}^{\text{T}})^{-1}$ (4)

$\mathbf{D}_{i+1}$ can thus be written as

$\begin{matrix} \mathbf{D}_{i+1}=(\mathbf{Y}_{i+1}\mathbf{W}_{i+1})(\mathbf{W}_{i+1}\mathbf{W}_{i+1}^{\text{T}})^{-1} \\ \text{=}\mathbf{D}_{i}-\mathbf{Y}_{i}\mathbf{W}_{i}^{\text{T}}\frac{(\mathbf{W}_{i}\mathbf{W}_{i}^{\text{T}})^{-1}\mathbf{w}_{i+1}\mathbf{w}_{i+1}^{T}(\mathbf{W}_{i}\mathbf{W}_{i}^{\text{T}})^{-1}}{\mathbf{w}_{i+1}^{T}(\mathbf{W}_{i}\mathbf{W}_{i}^{\text{T}})^{-1}\mathbf{w}_{i+1}+1} \\ +\mathbf{y}_{i+1}\mathbf{w}_{i+1}^{T}(\mathbf{W}_{i}\mathbf{W}_{i}^{\text{T}})^{-1}-\mathbf{y}_{i+1}\mathbf{w}_{i+1}^{T}\frac{(\mathbf{W}_{i}\mathbf{W}_{i}^{\text{T}})^{-1}\mathbf{w}_{i+1}\mathbf{w}_{i+1}^{T}(\mathbf{W}_{i}\mathbf{W}_{i}^{\text{T}})^{-1}}{\mathbf{w}_{i+1}^{T}(\mathbf{W}_{i}\mathbf{W}_{i}^{\text{T}})^{-1}\mathbf{w}_{i+1}+1} \end{matrix}$ (5)

Because $\mathbf{W}_{i}\in\mathbf{R}^{K\times i}$, $\mathbf{W}_{i}^{\text{T}}(\mathbf{W}_{i}\mathbf{W}_{i}^{\text{T}})^{-1}\in\mathbf{R}^{i\times K}$ can be expressed as

$\mathbf{W}_{i}^{\text{T}}(\mathbf{W}_{i}\mathbf{W}_{i}^{\text{T}})^{-1}=\left[\begin{matrix} \omega_{11}\text{ }\omega_{12}\text{ }\cdots\text{ }\omega_{1K} \\ \omega_{21}\text{ }\omega_{22}\text{ }\cdots\text{ }\omega_{2K} \\ \text{ }\vdots\text{ }\vdots\text{ }\ddots\text{ }\vdots\\ \text{ }\omega_{i1}\text{ }\omega_{i2}\text{ }\cdots\text{ }\omega_{iK}\text{ } \end{matrix}\right]$ (6)

Therefore, $\mathbf{D}_{i}$ can also be written as

$\begin{matrix} \mathbf{D}_{i}=\mathbf{Y}_{i}\mathbf{W}_{i}^{\text{T}}(\mathbf{W}_{i}\mathbf{W}_{i}^{\text{T}})^{-1} \\ \text{ }=\left[\mathbf{y}_{\mathbf{1}}\mathbf{ y}_{\mathbf{2}}\mathbf{ }\cdots\mathbf{ y}_{i}\right]\left[\begin{matrix} \omega_{11}\text{ }\omega_{12}\text{ }\cdots\text{ }\omega_{1K} \\ \omega_{21}\text{ }\omega_{22}\text{ }\cdots\text{ }\omega_{2K} \\ \text{ }\vdots\text{ }\vdots\text{ }\ddots\text{ }\vdots\\ \text{ }\omega_{i1}\text{ }\omega_{i2}\text{ }\cdots\text{ }\omega_{iK}\text{ } \end{matrix}\right] \\ \text{ }=\left[\sum_{r=1}^{i} \omega_{r1}\mathbf{y}_{r}\text{ }\sum_{r=1}^{i} \omega_{r2}\mathbf{y}_{r}\text{ }\cdots\text{ }\sum_{r=1}^{i} \omega_{rK}\mathbf{y}_{r}\right] \end{matrix}$ (7)

In (7), $\mathbf{D}_{i}$ is a series of linear combinations of training data, as is $\mathbf{D}_{i+1}$. As such, the learned dictionary must be a series of linear combinations of training data. When the noise signal itself is used as training data, the dictionary obtained using the dictionary learning algorithm is a series of linear combinations of noise signals, meaning that each atom in the learned dictionary is a combination of the third-order PPS with noise in multiple time periods if we use the noisy signal as training data.
